# Navigating the cancer trajectory in colorectal cancer: perceptions of cure over care, efficiency, and limited emotional support

**DOI:** 10.1007/s11764-026-02049-7

**Published:** 2026-06-02

**Authors:** Henriette Witte, Frede Donskov, Charlotte Handberg, Rasmus Krøijer, Lea Ladegaard Grønkjær

**Affiliations:** 1 Department of Oncology, University Hospital of Southern Denmark, Esbjerg, Denmark; 2 Department of Regional Health Research, University of Southern Denmark, Odense, Denmark; 3 National Rehabilitation Centre for Neuromuscular Diseases, Aarhus, Denmark; 4 Department of Public Health, Faculty of Health, Aarhus University, Aarhus, Denmark; 5 Department of Surgery, University Hospital of Southern Denmark, Esbjerg, Denmark; 6 Department of Gastroenterology, University Hospital of Southern Denmark, Esbjerg, Denmark

**Keywords:** Cancer survivorship care, Colorectal cancer, Compassion, Needs assessments

## Abstract

**Purpose:**

The aim of this study was to investigate interactions in the cancer trajectory of patients with colorectal cancer and their healthcare professionals, with a focus on needs assessment prior to cancer survivorship care.

**Methods:**

A qualitative ethnographic design guided by interpretive description was employed. Data were generated through 110 h of field observations at a surgical department and two semistructured focus group interviews with ten patients. Symbolic interactionism provided the theoretical lens. Data were analyzed inductively through coding, patterns, and thematic development.

**Results:**

The analysis generated three interrelated themes: *Perceptions on cure over care* highlighted that the healthcare system’s prioritization of biomedical outcomes often overshadowed emotional and psychosocial support. *Perceptions of communication shaped by efficiency* described the ways in which time constraints and fragmented care disrupted the relational continuity. *Perceptions of limited emotional support* illustrated the profound sense of abandonment that many patients experienced, particularly in the transition from active treatment to life after cancer.

**Conclusion:**

The study demonstrated that interactions around needs assessment are often shaped by structural and efficiency-driven priorities, leaving patients with limited emotional support. This highlights the critical need for further research aimed at embedding compassionate, patient-centered communication into needs assessment and survivorship care to ensure that patients’ psychosocial as well as biomedical needs are systematically addressed across the cancer trajectory.

**Implications for Cancer Survivors:**

A future holistic approach toward cancer survivorship care, where emotional and psychosocial well-being of cancer survivors is taken care of, to prevent feelings of abandonment and fear of recurrence.

## Introduction

Colorectal cancer is the third most common cancer worldwide and its prevalence continues to rise [1]. Advances in cancer treatment have significantly altered the cancer trajectory and improved the prognosis, which allows many patients diagnosed with colorectal cancer to survive and live longer [2]. As a result, patients experience a range of physical, psychological, and social needs [3, 4]. Consequently, the call to address these needs has increased to help patients return to a meaningful life [5, 6].

In Denmark, national cancer guidelines and five cancer plans have been developed to support coordinated cancer care across healthcare sectors [7, 8]. As part of these initiatives, formal needs assessments are intended to ensure systematic identification of patients’ physical, psychological, and social needs for cancer survivorship care in both hospital and community settings [9]. These assessments are designed to guide referrals to relevant cancer survivorship care programs, such as physical rehabilitation, psychosocial support, and supportive care interventions.

Numerous studies have shown the beneficial effects of cancer survivorship care [10, 11]. However, research indicates that despite the presence of formal structures, needs assessments are not consistently or systematically performed in clinical practice, and patients may therefore miss out on the benefits of cancer survivorship care [12]. When assessments do occur, they may focus primarily on biomedical symptoms rather than addressing broader emotional or psychosocial concerns that are important to patients [13]. This discrepancy between policy intentions and everyday clinical practice highlights the importance of exploring how needs assessment is enacted within interactions between patients and healthcare professionals.

Challenges related to the communication and interaction between patients and healthcare professionals during the cancer trajectory may explain why many patients do not receive cancer survivorship care [4, 13]. Effective communication and interaction are crucial for helping patients understand information and become actively involved in their cancer trajectory [14, 15].

The present study therefore investigates interactions in the cancer trajectory of patients with colorectal cancer, with a particular focus on how needs assessment prior to cancer survivorship care is experienced and performed in practice.

## Methods

This qualitative study employed an ethnographic fieldwork approach, combining participant observation (including observations, field notes, and informal conversations) with semistructured focus group interviews [16]. The study was guided by interpretive description as the methodological framework and symbolic interactionism as the theoretical foundation [16, 17].

## Design

Interpretive description guided the study by directing analytical attention toward clinically meaningful patterns in how needs assessment was enacted and experienced within everyday interactions across the cancer trajectory [16]. This approach is particularly valuable in complex clinical and social settings, in which practical implications matter [7]. Rather than aiming for theory generation, this approach enabled an in-depth exploration of patients’ experiences with a focus on producing practice-relevant knowledge that can inform improvements in cancer survivorship care. The analysis therefore centered on identifying recurring interactional patterns, tensions, and gaps between intended needs assessment practices and patients’ lived experiences.

Symbolic interactionism served as the theoretical lens for developing interview and observation guides as well as for the analysis. This sociological theory explores how individuals create meaning through social interaction, based on three key premises: (a) humans act toward things according to their ascribed meanings, (b) these meanings emerge from social interactions, and (c) they are continuously interpreted and modified through reflection [17]. In this study, particular attention was paid to how patients interpreted verbal and nonverbal communication with healthcare professionals, how meanings of being “done” with treatment were formed, and how these interpretations shaped patients’ understanding of their needs and available support.

Combining interpretive description with symbolic interactionism creates a strong qualitative approach for exploring complex human experiences [18, 19]. The integration of the two frameworks helps to understand how patients interpret and respond to illness, treatment, and interaction with healthcare professionals.

## Setting

This study was conducted at the Department of Surgery, University Hospital of Southern Denmark, Esbjerg. It was embedded in a larger randomized project investigating interventions designed to enhance needs assessment and support cancer survivorship care among patients with colorectal cancer. Patient follow-up was provided by surgeons and nurses during outpatient consultations. Needs assessment and discussions about cancer survivorship care were therefore embedded within interactions with surgeons and nurses. In Denmark, needs assessment prior to cancer survivorship care is supported by structured and validated tools developed at the regional level. These tools are designed as dialogue-based instruments to identify patients’ physical, psychological, social, and practical needs.

### Participants

The data for this study were generated through ethnographic fieldwork conducted by the first author between May and December 2024, comprising 110 h of field observation. During the fieldwork, the first author conducted participant observations and interacted with patients with colorectal cancer during their postoperative follow-up appointments at the outpatient clinic, the Department of Surgery, University Hospital of Southern Denmark, Esbjerg. This approach provided valuable insight into patients’ experiences and perspectives on their cancer trajectory by capturing how interactions and communication with healthcare professionals unfolded in routine clinical practice. The observations contextualized needs assessment processes and illuminated interactional dynamics that may not surface in interviews alone. Field notes were taken during observations and informal conversations.

In addition, two focus group interviews were conducted in June 2024 by the first author, with five participants in each group. In total, 12 patients with colorectal cancer were invited; however, two patients withdrew on the day of their respective focus group interviews, because of illness and cancer treatment. Therefore, 10 patients ended up participating, with five in each interview (Table 1). None of the participants reported receiving formal psychosocial support, such as psychological or social care from additional healthcare professionals, prior to the focus group interviews. This contextual factor is important for understanding the patients’ experiences of emotional support and needs assessment following completion of treatment.

**Table 1:** Characteristics of patients in focus group interviews (*N* = 10)

Sex (*n*, percent)	
Female Male	7 (70%) 3 (30%)
Age (mean, range)	65 (41–81 years)
Cancer diagnosis (*n*, percent)	
Colon cancer Rectal cancer	9 (90%) 1 (10%)
Chemotherapy after surgery (*n*, percent)	
Yes	5 (50%)
Cancer survivorship care (*n*, percent)	
Started or completed Pending	7 (70%) 3 (30%)
Civil status (*n*, percent)	
Married/cohabiting Single/divorced/widower	7 (70%) 3 (30%)
Occupation (*n*, percent)	
Working Retired	4 (40%) 6 (60%)

The focus groups provided in-depth insight into the patients’ experiences related to their cancer trajectory with a particular focus on needs assessment and the awareness of cancer survivorship care. The focus group interviews were conducted using a semistructured interview guide designed to progress from general experiences to more specific reflections on needs assessment and cancer survivorship care [17] (Table 2). The guide was reviewed and validated by a panel of six additional cancer patients who were not included in the focus group interviews. The purpose of this process was to ensure relevance, clarity, sensitivity, and meaningful patient involvement in the development of the interview questions. The guide was validated through a panel of six cancer patients to ensure the relevance and clarity of the questions and to guarantee meaningful patient involvement in the study. Initial questions focused on patients’ overall experiences with the surgical department and follow-up care, followed by questions addressing communication, involvement, and perceptions of needs assessment. This structure was intended to establish a safe group dynamic before addressing potentially sensitive topics.

**Table 2:** Interview guide (s*hortened for manuscript presentation*)

Introduction and information about the interview	- Purpose, timeframe, audio recording, GDPR, anonymity, consent - Use “one” instead of “I” when referring to other patients - Age, diagnosis, municipality
Experiences from the department	- How did you experience the surgical department? - Key questions or concerns? - Communication with doctors and nurses? - Your role in consultations before/after surgery? - Reflections and influences after discharge?
The needs assessment conversation	- How did you experience the conversation with the nurse? - What influenced your decision to rehabilitation? - Differences from the outpatient consultation? - Feeling of inclusion, being seen/heard? - Role of setting, relatives, and communication?
Cancer survivorship care	- How did the conversation influence your understanding/participation? - After-effects of illness (physical, psychological, social)? - What mattered in everyday life after cancer? - Reasons why some opt out of rehabilitation? - Importance in conversations with healthcare professionals?
Closing	- Thanks for sharing reflections and experiences - Opportunity for final comments or questions

The first author acted as moderator and actively facilitated balanced participation by inviting quieter patients to share their perspectives and by gently redirecting the discussion when individual patients began to dominate. Open-ended questions were supplemented with probing and follow-up questions to explore meanings, clarify statements, and encourage elaboration. Patients were encouraged to respond to each other’s experiences, while the moderator remained attentive to maintaining an inclusive and respectful discussion. Each focus group interview lasted for approximately 1 h and was digitally recorded and transcribed verbatim by the first author.

## Data analysis

The analysis was guided by the four iterative, inductive steps of analysis in interpretive description [12, 18, 19]. First, the data from the focus group interviews and field observation notes were transcribed into textual form. Second, the data was read and coded with a focus on identifying associations and patterns relevant to the aim of the study. Third, the data were reread and recoded. The first author conducted the primary coding and interpretive analysis of the data. To strengthen analytical rigor and credibility, the developing codes, categories, and thematic interpretations were continuously discussed within the author group, which included researchers with expertise in qualitative methodology, oncology, and cancer survivorship care. Consistent with interpretive description, the aim was not to establish interrater reliability in a positivist sense but to ensure interpretive coherence and analytic credibility through reflexive dialogue and collaborative interpretation across the research team. This iterative process involved comparing codes across data sources, collapsing overlapping codes, and grouping them into higher-level categories. These categories were then examined in relation to each other. This process strengthened analytic coherence and ensured that the final themes were grounded across both focus group interviews and field observations [16, 19]. This stage involved a critical appraisal of relationships within the data and an exploration of relevant thematic options, which led to the development of primary categorizations [16, 19]. Finally, the main messages were extracted from the data and shaped the final interpretation concerning patients’ experiences and perspectives of their cancer trajectory (from both observations and focus group interviews) [16, 19]. This led to a final categorization of themes, shaped in the way these themes were related and hierarchically correlated, so that the organizational structural factors affected and shaped the way healthcare professionals communicated and interacted with the patients [16, 19]. This was a back-and-forward process among the researchers, to ensure transparency and triangulation in the data generated, but also between the researchers [20]. We made sure that all the findings were consistent in all participants of the focus group interviews and within the field observations so that the themes were representative of all participants [20]. NVivo™ software, version 11, was used to support and manage data.

## Ethics

This study was conducted in accordance with the guidelines of the Helsinki Declaration. All participants received oral and written information, and written consent was obtained from all participants before the study began*.* The local health authorities approved the study (Journal number 22/42334) in accordance with the Danish Data Protection Agency law. No Ethics Committee approval was required due to Danish legislation.

## Results

**Fig. 1:**
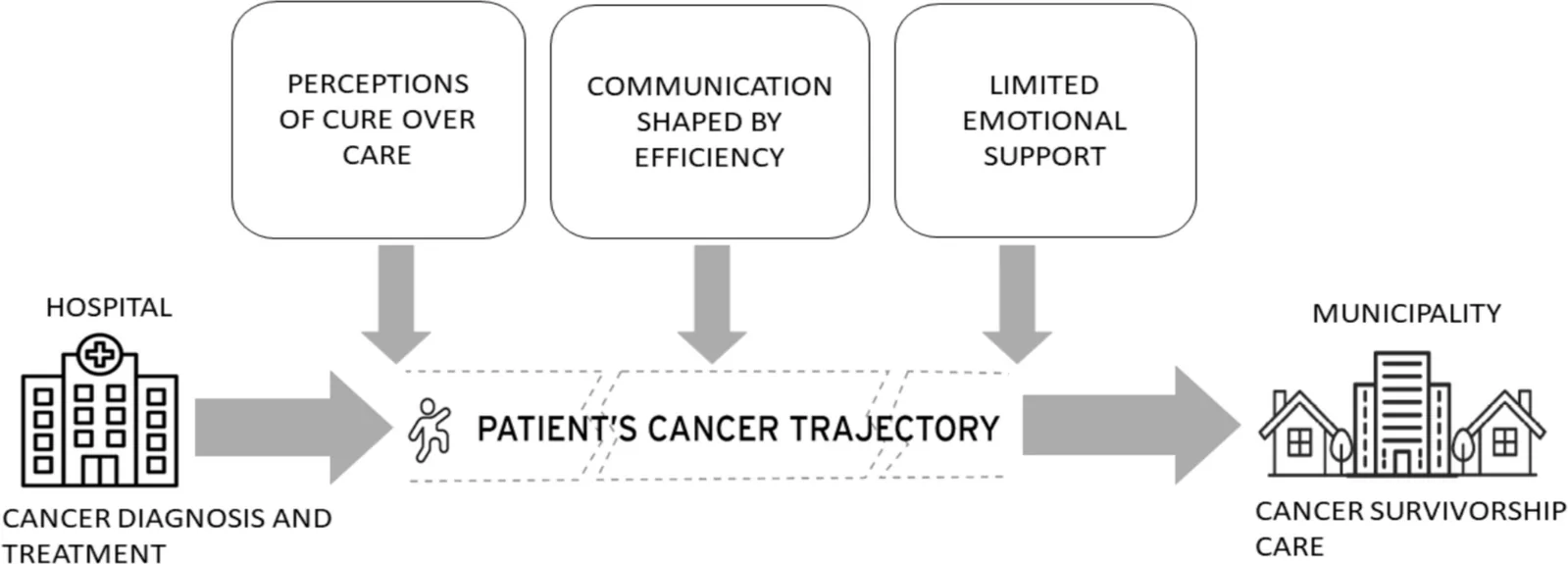
Impact of key themes on the colorectal cancer care trajectory. The figure illustrates how the three interrelated themes “cure over care,” “communication shaped by efficiency,” and “limited emotional support” affect patients’ cancer trajectory. Based on field observations and focus group interviews in the outpatient clinical setting following colon cancer surgery, it was evident that interactions around needs assessments were often shaped by structural and efficiency-driven priorities. This contributed to a fragmented care pathway, where limited emotional support complicated the assessment of patients’ needs across the entire trajectory, from hospital-based diagnosis and treatment to follow-up and survivorship support in the local community

The analysis explored the experiences of patients with colorectal cancer, focusing on their interactions with healthcare professionals. Three interrelated themes emerged: cure over care, highlighting how biomedical priorities often overshadowed emotional support; communication shaped by efficiency, showing how time constraints and fragmented care reduced patients’ sense of being acknowledged; and limited emotional support, reflecting feelings of abandonment, particularly during the transition from active treatment to life after cancer. Figure 1 provides an integrative overview of how these themes interact across the cancer trajectory, illustrating how structural and interactional factors jointly shape patients’ experiences of needs assessment and their transition to survivorship care.

### Perceptions on cure over care

Across observations and interviews, a dominant structural focus on biomedical outcomes over psychosocial care emerged clearly. This emphasis was manifested not only in the patients’ narratives but also in the daily routines and discourses of the healthcare professionals.

The healthcare professionals followed routines guided by checklists and standardized procedures. The encounters with patients were typically brief, task-oriented, and centered on specific clinical issues, such as information on lab results, surgery and treatment, or on treating isolated symptoms. The patients were moved efficiently through a predefined process with minimal dialogue about their broader situation or emotional needs. The focus was mainly on ensuring surgical success, which could result in patients being considered cancer-free, whereas there was less emphasis on supporting the person holistically.

The healthcare professionals appeared to work in a highly structured manner with a clear focus on documentation and task completion. The structural focus left a strong impression on the patients. From the patients’ perspective, the structural focus overshadowed other issues. One participant shared:

ID number 8:

*…because in my case, everything just happened so fast – I mean really fast. You knew nothing, right? You leave here, and then the following Monday you find out you have cancer, and then you’re having surgery, and everything is just spinning. I mean, the nurses at the department were great – you really can’t point a finger at anything – but maybe it would have been nice if there had been someone who knew a bit more… someone who could, what’s the word, spend half an hour or an hour just stopping by to talk about what was going on, because you have no idea! Everything is just moving ahead at full speed, and you can’t keep up at all! So, it might have been nice… someone saying, “This is what’s happening now, and yes, you might expect this or that”, you know?*

The structured, task-oriented approach left little room for the meaning-making processes that patients undergo in transitioning from an “ordinary person” to a “cancer survivor.” The lack of emotional scaffolding during and after surgery disrupted the patients’ identity reconstruction and reinforced a sense of emotional isolation. The patients struggled to interpret what it meant to be “done” with treatment when no one addressed their internal experience or psychosocial adaptation.

ID number 2:

*I was told I was a free man. But it left me with a hollow, empty feeling. When I walked out of there – of course, if I could, I would have lifted a little off the ground. But I felt empty… You walk away and then think: what now? I know I’m supposed to have a scope examination next year, and then again in five years…*

Meanwhile, the healthcare professionals, constrained by structural demands, rarely had the time, the space, or the systems in place to engage with the patients beyond clinical tasks. Moreover, although it was beneficial for multiple specialties and sectors to be involved in the patient’s cancer trajectory, there was room for improved coordination. Thus, the hospital provided no or limited information about the cancer survivorship care that was available through the local community setting. One patient stated:

ID number 4:

*If I hadn’t spoken to you, I think I would’ve felt much more on my own. However, because I talked to you and was referred to cancer survivorship care, I felt stronger. I think having had cancer is such a huge and horrible thing. And then they just say, “Well, now it’s gone – poof” (makes a sound and a hand gesture like someone being pushed along). “We’ll see you in a year”. And you’re left standing there with all the uncertainty and anxiety.*

Ultimately, this theme illustrated how a healthcare system structured around cure can inadvertently neglect care. Although it is essential to prioritize measurable biomedical outcomes, doing so leaves a gap in the emotional and relational support, particularly at critical transition points, which compromises the holistic well-being of patients navigating the cancer trajectory and neglects the benefits of cancer survivorship care.

### Perceptions of communication shaped by efficiency

In general, the patients had a positive view of the communication at the hospital; however, many observed that the healthcare professionals appeared extremely busy during outpatient appointments. The intense pace of work was evident even before the consultations began. The patients described seeing healthcare professionals pass by with hurried steps, often without making eye contact or offering a greeting, while they were waiting in the outpatient area. Rather than reacting negatively, many patients expressed sympathy for the healthcare professionals, noting how overwhelmed they seemed and acknowledging the pressures of the healthcare system.

Despite this understanding, many experienced the outpatient setting as impersonal and rushed. One common concern was the healthcare professionals’ failure to review the patient’s medical records before the appointment, which led to feelings of being undervalued or unseen. One participant explained:

ID number 4:

*Yeah and when I’ve been called in for scans and things like that, it’s been fine. But I’ve also been called in for outpatient consultations every time, so they could explain the results to me… but that part has been a bit inconsistent*…. *I’ve been told different things, and… well, when you show up and start explaining how you’re experiencing it – they say, “oh, I haven’t read that” – and then they have to read my medical record. There’s been a lack of preparation*. *I know things are busy. Yes, there’s a lot you can go in and read in a medical record, right? And they might miss something if they don’t read all of it! So yeah…*

These communication issues were further illustrated by observations documenting that the healthcare professionals often turned their backs on patients or focused on their computers during the interactions. Although it was presumably unintentional, such physical disengagement symbolically conveyed a lack of interest.

These micro-interactions played a central role in shaping the patients’ perceptions of their place within the healthcare system. When the professionals appeared unprepared or failed to acknowledge the patients’ emotional or informational needs, it disrupted their sense of connection and mutual understanding.

ID number 5:

*I felt completely alone in all of it. The doctors and nurses were kind, but they never really asked how I was doing mentally. It was only about the physical issues. However, I wasn’t prepared for how hard it would hit me afterwards. I thought I had the surgery, and then everything would be fine – but I completely broke down. I really sank into a dark place, and I thought… I wasn’t prepared for that! It would have been nice to hear other healthcare professionals say, “well, that’s very normal,” and then I could have thought – okay, that makes sense...*

Many participants expressed a strong need for support in navigating life after cancer. In outpatient settings, the focus often remained on biomedical outcomes and clinical follow-up; however, the patients longed for a more holistic attention to their emotional, physical, and practical needs. Several patients reported that cancer survivorship care was rarely introduced unless they themselves brought it up. One patient stated:

ID number 3:

*Well, maybe it should have been the staff in the outpatient clinic who was responsible for referring you further into the system, now that I think about it. Because otherwise, no one does anything! You’re left to handle it on your own. It might be a good idea if they took a moment to explain what kind of cancer survivorship care options are available – or simply asked how you’re doing… It’s a bit difficult when you come in for that consultation, because at that point you’re just focused on getting the results from the surgery. So, you don’t even think about what comes next. I think you really need someone to guide you for that next step afterwards. But I’m not even sure it’s their responsibility, right? Because they’re so busy. The next patient is already waiting to get their results. That’s why it’s much better to be picked up afterwards by someone like you. That’s my personal experience.*

Notably, many patients expressed uncertainty about the needs assessment prior to the cancer survivorship care. Several patients recalled that they had been contacted on the phone following their postoperative appointment and that they had not realized that this conversation was meant to assess their needs. Although needs assessments were intended to be conducted routinely as part of follow-up care, patients often did not perceive these interactions as formal or systematic assessments of their broader survivorship needs. In several cases, the conversations primarily focused on physical symptoms, bowel function, pain management, or practical treatment-related issues. Consequently, patients did not necessarily interpret these encounters as opportunities to discuss emotional, psychosocial, or existential concerns related to life after cancer. Some remembered receiving a need assessment form during their outpatient appointment but did not link it to a systematic needs assessment process. In these previous interactions, the focus had largely been on physical symptoms such as bowel movement frequency, stoma-related issues, or pain needs, which are prioritized by healthcare professionals as relevant to the cancer trajectory. These were not always the needs that the patients themselves felt were most urgent or for which they experienced an emotional need for support.

In sum, this theme illustrated how efficiency-driven communication limited the opportunities for emotional connection and individualized care. Although the information was often clear, the outpatient interactions felt rushed and impersonal, which left patients unsupported in navigating life after treatment. Many were unaware that needs assessments had taken place or misunderstood the purpose, as the focus remained on clinical symptoms rather than the patients’ own concerns. The constrained approach to communication risked overlooking the broader emotional and practical needs of cancer survivors.

### Perceptions of limited emotional support

One striking theme across the patients’ experiences was a pervasive sense of emotional isolation, particularly in the transition from active treatment to life after cancer. Although most of the patients felt well cared for medically, they consistently described a lack of emotional care and support during their hospitalization and an even more profound void following the conclusion of treatment.

The participants often described the experience of having to navigate the cancer trajectory largely on their own. Several noted that their emotional needs were not always fully acknowledged by the healthcare professionals, whose primary focus tended to be on the biomedical outcomes. This dynamic was also observed directly. During follow-up appointments, some patients became visibly emotional after difficult discussions. Although the clinical environment prioritized efficiency and task completion, a few healthcare professionals responded with subtle expressions of empathy, such as sustained eye contact or a reassuring touch. Nonetheless, the structured routines of clinical care appeared to offer limited opportunities for emotional connection.

The hospital functioned as a stage on which the patients’ enacted roles of illness, dependency, and recovery, affirmed through structured interactions with the healthcare professionals. When these interactions ended abruptly, the social and symbolic scaffolding that had upheld the patient identity dissolved as well. The sudden lack of follow-up or emotional inquiry left many patients in a liminal space: They were no longer patients but were not yet fully recovered or “whole.” The sense of abandonment was intensified by fears of recurrence and uncertainty about the future. As another participant shared:

ID number 1:

*I thought I would feel relieved when the treatment was over, but instead I just felt abandoned. Suddenly there were no follow-up appointments, no one to talk to – it felt like I was left to figure everything out on my own. I think it feels quite overwhelming. It seems like you hear a lot and read about so many people who have had cancer. I know several people with the same type of cancer, and then it comes back a few years later. They get cancer again. I find myself thinking a lot about that – “what if it comes back in a few years?” Did they really get everything when they removed it? That’s definitely something that lingers inside me... the thought of it coming back!*

These ongoing anxieties were not merely private but reflected a lack of external validation or interactional space to process postoperative fears. Without shared meaning-making, the participants internalized solitude and insecurity as key features of survivorship. One participant explained:

ID number 2:

*I think it feels very overwhelming. You hear a lot and read about so many people who’ve had cancer. I know several people with the same type of cancer, and then a few years later it comes back. They get cancer again. That’s something I think a lot about – “what if it comes back in a few years?” Did they really get everything when they removed it? That’s definitely something that sits with me… the fear that it might come back. And I also find it difficult that there’s such a long time between the follow-up exams – a scan now, and then not until five years from now before the colonoscopy. I had my final consultation last week, and no one even asked how I was really doing.*

Furthermore, many participants mentioned that although their families had the best intentions, they were not equipped to provide emotional support, partly because they also lacked guidance or help from the healthcare system. One patient described:

ID number 4:

*My family tried to be there for me, but they didn’t fully understand, and they also didn’t receive any help in dealing with it.*

This limited the patients’ ability to share their needs openly, which deepened their sense of emotional isolation. Importantly, some patients noted that brief interventions offered meaningful moments of mutual recognition that helped restore the symbolic coherence to the patients’ postoperative identities. For many, even small gestures of empathy from a healthcare professional carried significant emotional weight and contributed to a sense of being seen, supported, and emotionally met during a vulnerable phase of recovery. As another participant shared:

ID number 4:

*With you, it was more about the person – about how the whole process had been and what I needed to feel like myself again, in a way. I thought it was a good conversation, and you were very empathetic in the way you approached me as a patient. I was also contacted by cancer rehabilitation the following week, and that was because you had reached out to them. So, I thought that was really great.*

Ultimately, this theme revealed that the transition from treatment to everyday life after cancer was marked by emotional isolation and a lack of psychological support. Although the patients felt physically cared for, many struggled to make sense of their experiences without spaces for dialogue or validation of their emotional needs. The abrupt ending of structured care left the patients in a liminal state as no longer ill but not yet whole, which amplified their fears of recurrence and uncertainty about the future. Although they were rare, moments of genuine emotional recognition were deeply valued and helped re-establish a sense of coherence and support.

## Discussion

This study provides an in-depth exploration of how interactions between patients with colorectal cancer and healthcare professionals shape experiences of needs assessment across the cancer trajectory. It revealed three interconnected themes that captured essential aspects of their trajectory. Together, these findings point to the critical need to integrate patient-centered care as a core component throughout the entire cancer trajectory.

The findings demonstrate that a strong organizational emphasis on biomedical outcomes and efficiency continues to shape clinical encounters, often at the expense of emotional and psychosocial support. While standardized pathways and task-oriented routines are designed to ensure efficiency and safety, our findings extend existing research by illustrating how patients experience these structures as emotionally distancing, particularly at moments when meaning-making and reassurance are most needed. Previous studies have shown that standardized cancer pathways can marginalize relational aspects of care [12, 21]. Our study adds to this knowledge by demonstrating how these priorities are enacted in everyday interactions and how they shape patients’ interpretations of being “done” with treatment, often leaving them uncertain and emotionally unsupported. The barriers to addressing patients holistically may include time constraints, communication limitations, and a lack of training in compassionate or emotional support [22]. The patients in this study expressed a strong need for someone to talk to about the broader meaning of their illness and recovery, a need that remained largely unmet within the current approach at the hospital. The findings reinforce the call for more holistic models of care that integrate attention to patients’ emotional and psychosocial well-being alongside clinical outcomes [23].

The communication during outpatient follow-up was perceived as efficient but impersonal. The patients acknowledged the heavy workloads faced by the healthcare professionals but nevertheless described a sense of being rushed through the consultations. One key concern was the lack of preparation by some healthcare professionals, such as not reviewing medical records before the consultation. This disrupted the continuity and left the patients feeling undervalued. Importantly, several patients were unaware that they had participated in a needs assessment or misunderstood its purpose, which indicates that these assessments were implemented too superficially or without proper explanation. These findings demonstrate the ways in which efficiency-driven systems can reduce opportunities for meaningful dialogue and shared understanding. Although the information given was often medically accurate, it lacked the emotional resonance and person-centered attention needed for the patients to feel supported [24, 25]. As noted in prior research, constrained availability and brief encounters can significantly disrupt the patients’ sense of acknowledgment and continuity [22].

A particularly salient contribution of this study is the insight into patients’ emotional isolation following the completion of active treatment. Consistent with earlier research, emotional needs were often unaddressed unless explicitly raised by patients themselves [26]. However, our findings further demonstrate how the abrupt withdrawal of structured care contributes to a liminal state in which patients feel neither ill nor fully recovered. Through the lens of symbolic interactionism, this can be understood as a breakdown in shared meaning-making, where the absence of interactional support leaves patients to interpret fears of recurrence and uncertainty in isolation. Even brief moments of empathic engagement were described as highly significant, underscoring the relational nature of emotional support in survivorship care. Addressing such needs requires healthcare professionals to demonstrate strong communication skills and interpersonal qualities, including active listening, genuine interest, and respect [24]. Compassionate communication has been shown to improve treatment adherence, reduce anxiety, and alleviate distress among patients with cancer [25]. Compassion involves recognizing suffering and responding with a genuine intention to alleviate it, and importantly, such skills can be strengthened through targeted training [27, 28]. Our findings support the growing body of evidence that compassion should not be viewed as an individual trait alone but as a relational and organizational practice that must be supported by appropriate structures and resources.

The findings can be further interpreted using the Medical Research Council (MRC) framework for complex interventions [29, 30]. From this perspective, needs assessment constitutes a complex intervention whose effectiveness depends on alignment between theoretical intent, contextual conditions, and implementation practices. Our study highlights how needs assessments, when treated primarily as administrative tasks, lose their potential to support meaningful engagement with patients’ psychosocial needs. The findings suggest that for needs assessments to be effective, they must be better integrated into routine clinical practice, adapted to local contexts, and supported by sufficient time and relational continuity. This implies that future interventions should not only focus on introducing tools or forms but also address organizational priorities, professional roles, and communication practices that shape how needs assessments are enacted.

From a system’s perspective, improving psychosocial care following cancer treatment requires changes at multiple levels. In Denmark, psychosocial support for cancer survivors is provided through a combination of hospital-based services, municipal rehabilitation programs, and nongovernmental organizations. Although specialized psychosocial providers such as psychologists, social workers, and rehabilitation professionals are available and services are publicly funded, access and implementation vary across settings and often depend on referral practices, local resources, and patients’ ability to navigate the system.

Our findings suggest that clearer integration of needs assessment into routine clinical workflows could function as a critical entry point to psychosocial support. When needs assessments are conducted as meaningful, dialogical processes rather than administrative tasks, they may facilitate timely identification of emotional and psychosocial needs and improve coordination between hospital-based and community-based services. This suggests that meaningful survivorship care cannot rely on policy frameworks or guideline implementation alone but requires organizational and relational conditions that enable healthcare professionals to engage in sustained, person-centered, and emotionally responsive follow-up throughout the cancer trajectory. Although national guidelines and cancer plans emphasize psychosocial care and rehabilitation as integral components of survivorship care, our findings indicate that guidelines alone are unlikely to resolve existing gaps unless they are supported by structures that facilitate compassionate communication, continuity of care, and action on identified patient needs [8, 9].

The findings therefore point toward a need for survivorship care models that strengthen continuity, emotional support, and relational follow-up across the transition from active treatment to survivorship. Future initiatives should focus on integrating needs assessments as meaningful, dialogue-based encounters embedded within routine clinical practice rather than as isolated administrative tasks. This may include clearer responsibility for survivorship follow-up, improved coordination between hospital and community services, and greater attention to fear of recurrence and psychosocial distress among cancer survivors and their families. Furthermore, healthcare professionals may benefit from communication and compassion-focused training to support more person-centered and emotionally responsive interactions. However, the effectiveness of such initiatives depends on organizational conditions, including sufficient time allocation, continuity of care, and clearly defined professional responsibilities. Together, these findings highlight the importance of aligning policy ambitions with everyday clinical practice to ensure that psychosocial support becomes a consistent and accessible component of cancer survivorship care.

## Limitations

This study has several limitations that should be considered when interpreting the findings. First, the study was conducted at a single surgical department within the Danish healthcare system, which may limit the transferability of the findings to other organizational and cultural contexts. While qualitative research does not aim for statistical generalizability, the findings should be understood in relation to the specific structural and policy conditions of the Danish setting. Second, the sample size was relatively small, consisting of ten patients participating in two focus group interviews, complemented by field observations. Although this design enabled rich, in-depth insights, it may not capture the full diversity of experiences among patients with colorectal cancer. The focus group format may also have influenced how experiences were expressed, as some patients may have been less inclined to share personal or emotionally challenging perspectives in a group setting or may have aligned their responses with those of other patients. While the moderator actively encouraged balanced participation and invited alternative viewpoints, it cannot be ruled out that some patients’ experiences remained underexplored. Notably, although there was broad agreement across themes, variations in how patients described emotional support and coping strategies were observed, indicating that experiences were not entirely uniform. Third, the first author conducted both the field observations and the focus group interviews, which may have influenced data generation and interpretation. Reflexive practices and systematic analytic procedures were applied to mitigate potential bias; nevertheless, the dual role of observer and interviewer should be acknowledged. Finally, the study focused primarily on hospital-based experiences within a limited timeframe. Perspectives from healthcare professionals and longer-term survivorship experiences were not included and should be explored in future research. Despite these limitations, the use of ethnographic fieldwork combined with focus group interviews provided valuable insights into how patients experienced needs assessment and survivorship care, and the consistency of findings across data sources strengthens the credibility of the study.

## Conclusion

In conclusion, this study demonstrates that interactions around needs assessment in colorectal cancer care are frequently shaped by structural and efficiency-driven priorities, leaving patients with limited emotional support during critical transitions. These findings underscore an urgent need for action to ensure that needs assessments function as meaningful opportunities for engagement rather than as administrative tasks. Addressing these gaps requires healthcare professionals to integrate compassionate, patient-centered communication into routine practice and to actively engage with patients’ psychosocial as well as biomedical needs. At a policy level, organizational and structural conditions must support such practices by allowing sufficient time, continuity, and contextual adaptation of needs assessments across the cancer trajectory. Without such changes, patients may continue to experience survivorship as a period marked by emotional uncertainty, limited psychosocial support, and fragmented transitions between treatment and everyday life.

## Data Availability

This study was conducted in accordance with the guidelines of the Helsinki Declaration. All participants received oral and written information, and written consent was obtained from all participants before the study began. The local health authorities approved the study (Journal number: 22/42334) in accordance with the Danish Data Protection Agency law. No Ethics Committee approval was required due to Danish legislation.
